# Whole-Genome Analysis of Antimicrobial-Resistant *Salmonella enterica* Isolated from Duck Carcasses in Hanoi, Vietnam

**DOI:** 10.3390/cimb45030143

**Published:** 2023-03-08

**Authors:** Trung Thanh Nguyen, Hoa Vinh Le, Ha Vu Thi Hai, Thanh Nguyen Tuan, Huong Minh Nguyen, Da Pham Xuan, Huyen Tran Thi Thanh, Hao Hong Le Thi

**Affiliations:** 1 Department of Food Microbiology and Genetically Modified Food, National Institute for Food Control, Cau Giay, Hanoi 100000, Vietnam; 2 Division of Bacteriology, Department of Infection and Immunity, School of Medicine, Jichi Medical University, Tochigi 329-0498, Japan; 3 Center for Genetic and Reproductive Health, Faculty of Medicine, Vietnam National University, Ho Chi Minh City 700000, Vietnam; 4 Vinmec Research Institute of Stemcell and Gene Technology, Hai Ba Trung, Hanoi 100000, Vietnam

**Keywords:** whole-genome sequencing, *Salmonella*, antimicrobial resistance, virulence plasmid, salmonellosis, duck carcass

## Abstract

*Salmonella enterica* is one of the most dangerous foodborne pathogens listed by the World Health Organization. In this study, whole-duck samples were collected at wet markets in five districts in Hanoi, Vietnam, in October 2019 to assess their *Salmonella* infection rates and evaluate the susceptibility of the isolated strains to antibiotics currently used in the prophylaxis and treatment of *Salmonella* infection. Based on the antibiotic resistance profiles, eight multidrug resistance strains were whole-genome-sequenced, and their antibiotic resistance genes, genotypes, multi-locus sequence-based typing (MLST), virulence factors, and plasmids were analyzed. The results of the antibiotic susceptibility test indicate that phenotypic resistance to tetracycline and cefazolin was the most common (82.4%, 28/34 samples). However, all isolates were susceptible to cefoxitin and meropenem. Among the eight sequenced strains, we identified 43 genes associated with resistance to multiple classes of antibiotics such as aminoglycoside, beta-lactam, chloramphenicol, lincosamide, quinolone, and tetracycline. Notably, all strains carried the *bla*_CTX-M-55_ gene, which confers resistance to third-generation antibiotics including cefotaxime, cefoperazone, ceftizoxime, and ceftazidime, as well as resistance genes of other broad-spectrum antibiotics used in clinical treatment such as gentamicin, tetracycline, chloramphenicol, and ampicillin. Forty-three different antibiotic resistance genes were predicted to be present in the isolated *Salmonella* strains’ genomes. In addition, three plasmids were predicted in two strains, 43_S11 and 60_S17. The sequenced genomes also indicated that all strains carried SPI-1, SPI-2, and SPI-3. These SPIs are composed of antimicrobial resistance gene clusters and thus represent a potential threat to public health management. Taken together, this study highlights the extent of multidrug-resistant *Salmonella* contamination in duck meat in Vietnam.

## 1. Introduction

Foodborne pathogens such as non-typhoid *Salmonella* are of great concern globally [1,2,3]. According to the European Food Safety Authority (EFSA), *Salmonella* alone induces over 91,000 foodborne salmonellosis cases each year, resulting in an economic burden of human salmonellosis reaching up to EUR 3 billion per year. In the US, *Salmonella* is the cause of 1.35 million infections, 26,500 cases of hospitalization, and 420 deaths annually [4]. As a result, *Salmonella* infections continue to be a global concern, with millions of cases reported every year, which creates difficulty in identifying the sources and causal organisms involved. Within the *Salmonella* genus, *Salmonella enterica* is the leading factor that causes foodborne outbreaks. This species consists of six subspecies: enterica, arizonae, diarizonae, salamae, houtenae, and indica; an estimated 2659 serovars have been identified to date. Moreover, *S. enterica* subspecies *enterica* has 1547 serovars, of which 99% are infectious to humans and animals [5].

Data collected from around the world have shown that chicken meat and chicken egg are the main sources of *Salmonella* infection [3,5,6]. Today, with the increase in the human consumption of poultry, duck is raised in industrial conditions as chicken. Consequently, the risk of exposure to antimicrobial resistance (AMR) genes is the same as that for chickens [7]. Tran et al. observed that 8.7% of duck fecal samples in the Mekong delta region were positive for *Salmonella* [8]. In Korea, research in 2013 revealed that the rate of *Salmonella* infection in duck flocks was 43.4% [9]. In recent years, the antibiotic resistance characteristics of *Salmonella* have become a significant concern for the public [4,10,11,12,13]. In low- and middle-income countries, antimicrobials are usually used in large amounts to treat and prevent bacterial infections, increasing productivity in animal farming [14,15,16]. We have previously found that domestic animals, such as pigs, chickens, and ducks, from the Mekong Delta harbor *Salmonella* at a high rate [17]. It is therefore important to determine the rate of *Salmonella* contamination also in retail meat, since meat that originates from domestic animals has been known to be an important source of human infection by *Salmonella* [18]. This can be a serious problem, especially in the countryside where there is little knowledge about the hygienic handling of meat and no refrigeration. However, inappropriate antibiotic use in agricultural and veterinary practices has resulted in the emergence of multidrug-resistant (MDR) bacteria and transferrable genetic loci, which together represent a serious worldwide public health problem, particularly the spread of pathogens and antimicrobial resistance genes (ARG) [19]. A recent study on the prevalence of endemic *Salmonella* in raw meat collected from traditional markets in Ho Chi Minh City (Vietnam) revealed that 37.89% of all isolated strains were resistant to at least one antibiotic, and up to 8.7% of strains were resistant to more than six antibiotics [20]. In addition, Tsai and Hsiang’s research in Taiwan indicated that 40% of *Salmonella* strains isolated from duck were resistant to ampicillin, chloramphenicol, tetracycline, and sulfamethoxazole/trimethoprim [21]. Little is known, however, about the prevalence of *Salmonella* in duck meat and duck production in Vietnam.

Although several studies of MDR *Salmonella* in animal-derived products have been published, data on duck meat contamination in Vietnam are still lacking. There have been several traditional molecular-typing approaches used to explore the subsequence transmission of antibiotic-resistant *Salmonella* in humans, animals, and the environment, including pulsed-field gel electrophoresis (PFGE) [22] and multi-locus sequence-based typing (MLST) [23]. However, the disadvantage of these approaches seems to be a lack of the discriminating power needed to separate closely related *Salmonella* isolates in epidemic investigations and to distinguish between intraserovar isolates from different hosts. In order to obtain a better understanding of antimicrobial-resistant pathogen molecular epidemiology, the application of whole-genome sequencing (WGS) has significantly influenced research in recent times [24]. Numerous projects utilizing WGS have been reported in comprehensive studies on foodborne pathogens, especially those in foodborne outbreaks, while also giving more insight about controlling antimicrobial resistance [24,25,26,27]. Pornsukarom et al. reported that multiple virulence genes were identified among the several *Salmonella* serovars across different sources based on WGS [28]. These genes were associated with various essential *Salmonella* transmission and infection mechanisms, including adhesion, the type III secretion system (T3SS), and host recognition/invasion [28]. Ultimately, due to the lack of utilization of WGS in the genomic research of *Salmonella* serovars distributed in Vietnam, this study aims to analyze the rate of *Salmonella* infection and identify ARG in various serovars collected from infected duck samples.

## 2. Materials and Methods

### 2.1. Sample Collection

A total of 47 duck carcass samples were collected in wet markets during October 2019 from five districts in Hanoi, Vietnam, including Ba Dinh (n = 8), Cau Giay (n = 3), Dong Da (n = 12), Hoang Mai (n = 11), and Thanh Xuan (n = 13). For each sample, a whole duck was purchased and individually placed in a sterilized plastic bag. All samples were preserved in sample transport containers filled with dry ice and sent to the laboratory within the same day for analysis.

### 2.2. Salmonella Isolation

Isolation of *Salmonella* was performed according to the United States Department of Agriculture (USDA) standard methods for rinsing whole-bird samples [29]. Each sample was aseptically placed in a sterile plastic bag containing 400 mL of buffered peptone water (BPW, Difco, Detroit, MI, USA). The whole duck was then rinsed by shaking for 2 min. Next, 30 mL rinsed fluid of each sample was vortex-mixed in 30 mL of BPW for 15 s, followed by incubation at 37 °C for 18–24 h. Then, 0.1 mL portions of BPW enrichment broth were transferred to 10 mL of Rappaport–Vassiliadis broth (RV; BD, USA), and 0.5 mL portions subjected to pre-enrichment were transferred into 10 mL of tetrathionate broths (TT; BD, Franklin Lakes, NJ, USA) and continued to be incubated for 24 h at 41.5 °C. The selective cultures were streaked on xylose–lysine–desoxycholate agar (XLD; BD, Franklin Lakes, NJ, USA) and bismuth sulfite agar (BSA; BD, Franklin Lakes, NJ, USA) plates, followed by incubation at 37 °C for 24 h. Typical colonies were selected for biochemical tests, and polyvalent antisera for O and H antigens (BD, Franklin Lakes, NJ, USA) in order to identify *Salmonella* isolates. *Salmonella* ATTC 14028, *Salmonella* ATCC 13076, and *Escherichia coli* ATCC 8389 were used as the quality control standards for the isolation procedure. All isolated *Salmonella* strains were stored at −80 °C for further analyses [30].

### 2.3. Antibiotic Susceptibility Test

The antimicrobial susceptibility of each *Salmonella* isolate was tested using the disk diffusion method, which was introduced by Bauer and Kirby in 1956 [31]; then, criteria for the classification of each MDR strain were followed according to Clinical and Laboratory Standards Institute (CLSI) guidelines (Wayne, PA, USA) [32]. Antibiotic susceptibility was determined using Liofilchem discs (Roseto degli Abruzzi (TE), Italy) with the following antibiotics: cefuroxime (CXM, 30 µg); ceftriaxone (CRO, 30 µg); cefoxitin (FOX, 30 µg); cefazolin (CZ, 30 µg); cefotaxime (CTX, 30 µg); ceftazidime (CAZ, 30 µg); an ESBL disc kit (acc. to CLSI) containing cefotaxime (CTX, 30 µg), cefotaxime + clavulanic acid (CTL, 30 + 10 µg), and ceftazidime (CAZ, 30 µg); ceftazidime + clavulanic acid (CAL, 30 + 10 µg); an AmpC disc kit containing cefotaxime (CTX, 30 µg), cefotaxime 30 μg + cloxacillin (CTC), and ceftazidime (CAZ, 30 µg); ceftazidime 30 μg + cloxacillin (CAC); gentamicin (CN, 10 µg); tetracycline (TE, 30 µg); ciprofloxacin (CIP, 5 µg); chloramphenicol (C, 10 µg); ampicillin (AMP, 10 µg); meropenem (MRP, 10 µg); imipenem (IMI 10 µg); nalidixic acid (NA, 30 µg); and trimethoprim (TM, 5 µg). 

Briefly, *Salmonella* was grown in Tryptic Soy Agar plates (TSA; BD, Franklin Lakes, NJ, USA) overnight, and a suspension with a concentration of 1.0 × 10^6^ cfu/mL was prepared. We then used a sterile cotton swab to evenly spread the bacterial suspension on Mueller Hinton agar plate. Next, we placed antibiotic disks on the surfaces of the inoculated and dried plates and incubated the plates in an inverted position at 37 °C for 16–18 h. *Escherichia coli* (ATCC 25922) was used as the quality control standard. *Salmonella* spp. that showed resistance to more than three classes and more than one antibiotic in a single class was designated as the MDR strain.

Antibiotic susceptibility test was repeated three times; only average results are shown.

### 2.4. Genomic DNA Extraction, Whole-Genome Sequencing, and De Novo Assembly

In total, 8 strains were selected for whole-genome sequencing (WGS) based on the antibiotic resistance profiles among the 34 tested isolates. Genomic DNA was extracted from 1 mL of an overnight culture grown in Brain Heart Infusion broth (BHI; BD, Franklin Lakes, NJ, USA) using a PureLink™ Genomic DNA Mini Kit (Invitrogen, Thermofisher scientific, Waltham, MA, USA) according to the manufacturer’s protocol. A library was prepared for sequencing, and WGS sequencing was performed using an Illumina MiSeq system (Illumina, San Diego, CA, USA), as described by the respective manufacturers.

Read trimming was carried out using Trimmomatic tool (v 0.32, Julich, Germany) to remove Nextera adapter sequence and poor-quality basecalls [33]. Quality control was conducted with FastQC (v 0.11.9) (https://www.bioinformatics.babra-ham.ac.uk/projects/fastqc/, accessed on 1 July 2020). De novo assembly was performed using SPAdes (v 3.15.3, Saint Petersburg, Russia) [34]. Contigs were ordered against the *Salmonella enterica* sbsps. *enterica* serovar Typhimurium strain ATCC 14028 using ABACAS v1.3.1 (Austin, TX, USA) with the -dmbc setting [35].

### 2.5. Annotation

The raw sequenced reads were analyzed using the *Salmonella* In Silico Typing Resource for serovar identification [36]. The assembled contigs were screened for ARG using Abricate [37], plasmid replicons, and virulence genes. The antibiotic resistance genes were determined by screening the draft genome against ResFinder [38], the Comprehensive Antibiotic Resistance Database (CARD) [11], and the Antibiotic Resistance Gene-ANNOTation (ARG-ANNOT) [39] database. The search for plasmid replicons was performed by screening the draft genome against the PlasmidFinder database [40]. The presence of virulence genes was identified using Virulence Factor Database (VFDB) [41]. Mobile genetic elements were detected using the mobile element finder tool [42]. MLST profiles were determined using the software package MLST v2.16.1 from the draft assemblies [23].

## 3. Results

### 3.1. Prevalence of Salmonella spp.

The positive rate of *Salmonella* in the 47 duck carcass samples was 72.34% (34/47 samples), in which the infection rate was different district-wise, with the highest infection rate found in the Cau Giay district (100%, 3/3 samples), followed by the Dong Da district (75.00%, 9/12 samples), Hoang Mai district (72.73%, 8/11 samples), Thanh Xuan district (69.23%, 9/13 samples), and Ba Dinh district (62.50%, 5/8 samples).

The results of the core-genome multi-locus sequence typing (cgMLST) analysis showed that the MDR *Salmonella* strains isolated from different areas were clustered into different sequence types and phenotypically different in terms of serovars, serogroups, and the presence of H and O antigens (Table 1).

Within these eight isolates, two MLSTs were identified, of which seven were classified as MLST 321. These seven isolates were identified as the serovar Muenster based on the presence of O antigens 3,{10}{15}{15,34} and H antigens i, 1, 5. Muenster was also the most prevalent serovar in this study. Another serotype found in this study was Kentucky (n = 1).

### 3.2. Antibiotic Resistance Profiles of the Salmonella Isolates

**Figure 1:**
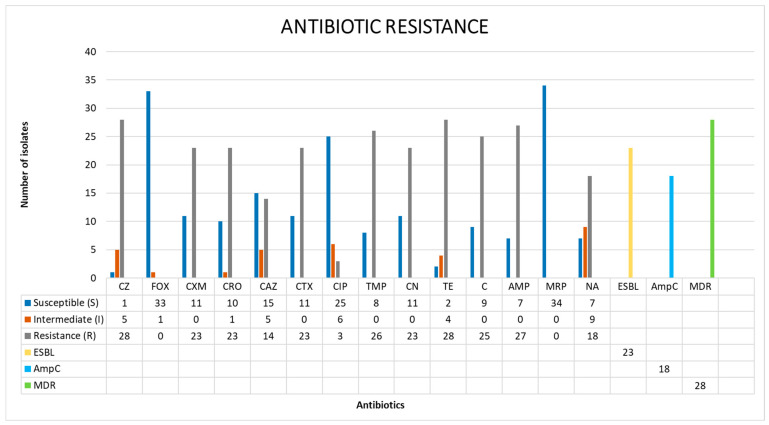
Antibiotic resistance profiles of duck carcass samples: cefazolin (CZ), cefoxitin (FOX), cefuroxime (CXM), ceftriaxone (CRO), ceftazidime (CAZ), cefotaxime (CTX), ciprofloxacin (CIP), trimethoprim (TMP), gentamicin (CN), tetracycline (TE), chloramphenicol (C), ampicillin (AMP), meropenem (MRP), nalidixic acid (NA), extended-spectrum beta-lactam (ESBL), AmpC β-lactamase (AmpC), and multidrug resistance (MDR).

The antibiotic resistance profiles of all isolates are shown in Figure 1. Among the 34 *Salmonella* strains isolated and tested in this study, 97.06% (33/34 strains) presented a resistance phenotype to at least one of the 15 antimicrobials in the tested panel (Table A1).

The results of the antibiotic susceptibility test indicated that phenotypic resistances to tetracycline and cefazolin were the most common (82.35%, 28/34), followed by ampicillin (79.41%, 27/34), trimethoprim (76.47%, 26/34), chloramphenicol (73.53%, 25/34), cefuroxime (67.65%, 23/34), ceftriaxone (67.65%, 23/34), cefotaxime (67.65%, 23/34), gentamicin (67.65%, 23/34), nalidixic acid (52.94%, 18/34), ceftazidime (41.18%, 14/34), and ciprofloxacin (8.82%, 3/34). However, all isolates were susceptible to cefoxitin and meropenem (Figure 1).

Among the 34 isolates, 18 (52.94%) had the ability to synthesize the AmpC β-lactamase enzyme, and 67.65% of all tested strains were identified as ESBL strains (23/34). In total, 28/34 strains (82.35%) were considered multidrug-resistant strains.

#### 3.2.1. Whole-Genome Sequencing and Genome Characteristics

For a better understanding of genotypic antibiotic resistance, eight strains were whole-genome sequenced using the Illumina platform, including isolate numbers 31_S7, 42_S10, 43_S11, 45_S12, 51_S13, 57_S16, 60_S17, and 68_S20. After de novo assembly using the SPAdes algorithm, the number of contigs was found to range from 305 to 493 contigs, while the N50 values ranged from 22,011 to 36,997 bp. The GC (%) content of the genomes ranged from 52.15% to 52.51% (average 52.36%). All sequencing data were deposited in Genbank with the SRA accession numbers listed below (Table 2).

#### 3.2.2. Antibiotic Resistance Gene Profile

Using in silico prediction, the sequenced genomes of MRD isolates were predicted to carry 43 different ARG in total (Table 3 and Table A2), all of which belong to different gene families. Genotypic predictions of those ARM genes fully matched the phenotypic results yielded in the antibiotic susceptibility test above.

##### Aminoglycoside

All eight strains were resistant to gentamicin, which is an antibiotic belonging to the aminoglycoside class. The sequenced genomes carried a diverse range of aminoglycoside resistance genes. Among these, coding genes for aminoglycoside acetyltransferase (*aac(3)-Iia, aac(3)-IId_1*, *aac(6)-Iaa_1*, and *aac(6)-Iy*), a protein frequently found in *S.* Enteritidis and *S.* Enterica, were identified in all of the sequenced isolates. Genes that confer resistance to aminoglycoside also included *ant(3)-Ia_1*, *aadA17, aadA22,* and *aadA7_1*; these genes encode for aminoglycoside nucleotidyltransferase (found in all eight strains). In the *aph group*, *aph(3)-Ia_3* and *aph(6)-Id_1* were found to encode for aminoglycoside phosphotransferases (found in six strains). Another important gene, *rmtB-1,* was detected in isolate 68_S20; this gene encodes for 16S rRNA methyltransferase.

##### Beta-Lactam

The antibiotic susceptibility results indicated that all eight isolates were resistant to third-generation cephalosporins. These phenotypes were later confirmed to feature alongside beta-lactam resistance-related genes. *bla*_CTX-M-55_ is a notable gene in this group involved in resistance to various antibiotics, including amoxicillin, ampicillin, aztreonam, cefepime, cefotaxime, ceftazidime, ceftriaxone, piperacillin, and ticarcillin. In addition, seven out of eight isolates were predicted to carry the gene *bla*_TEM-1B_1_. Some isolates carried several beta-lactam resistance genes. In particular, isolate 68_S20 had one contig that predicted five genes (*bla*_CTX-M-55_, *bla*_TEM-1B_, *bla*_TEM-206_, *bla*_TEM-141_, and *bla*_TEM-214_), and isolate 51_S13 carried 11 beta-lactam resistance-related genes in total, including *bla*_LAP-2_, *bla*_TEM-214_, *bla*_TEM-206_, *bla*_TEM-33_, *bla*_TEM-1B_, *bla*_TEM-216_, *bla*_TEM-209_, *bla*_CTX-M-55_, *bla*_TEM-34_, *bla*_TEM-210_, and *bla*_TEM-141_. Notably, isolate 51_S13 featured a contig predicted to contain the beta-lactam resistance genes mentioned above, excluding *bla*_CTX-M-55_.

##### Quinolone

In eight ciprofloxacin and nalidixic acid phenotypically resistant strains, seven out of them contained the *qnrS1_1* gene, which is involved in the mechanism of quinolone resistance. The mutations associated with quinolone resistance were as follows: strain No. 68_S20 was related to four mutations, including the *parC:* p. T57S mutation (ACC to AGC mutation encoding the amino acid T to S); *parC*:p.S80I (AGC to ATC mutation encoding the amino acid S to I); the *gyrA*:p.S83F mutation (TCC to TTC mutation encoding the amino acid S to F); and the *gyrA*:p.D87N mutation (GAC to AAC mutation encoding the amino acid D to N). Seven out of eight strains carried the *parC*:p.T57S mutation. Furthermore, in one sample (sample 31_S7), *qnrS_1* was found to be located on the insertion sequence ISKpn19, which belongs to the ISKra4 family.

##### Other Genes

We found that six out of eight strains carried the gene *floR-2*, which encodes for chloramphenicol acetyltransferase. Interestingly, the 5/6-sample fluorine-2 gene was observed in the insertion sequence ISVs3 of the IS91 family (five out of eight samples).

Only one strain carried the gene *mph(A)_2*, which encodes for the enzyme macrolide phosphotransferase. Seven out of eight strains contained the gene *tet(A)_6*, which is involved in resistance to the tetracycline group; four out of eight strains carried genes (*sul1_5* or *sul3_2*) related to resistance by replacing the antibiotic target of sulfonamide; and one out of eight isolates carried the gene *fosA3_1*, encoding the gene for osfomycin thiol transferase. These genes are involved in antibiotic resistance to osfomycin. The genomes of all eight isolates appeared to carry the *dfrA14_5* gene, this gene is involved in trimethoprim resistance through the formation of trimethoprim-resistant dihydrofolate reductase dfr. All eight strains contained the gene *arr-3_4*, which encodes for Rifampin ADP-ribosyltransferase. All eight strains contained the gene *lnu*(*F*)*_1* (equivalent to *lin*(*F*)), which is the gene that encodes for integron-mediated nucleotidyltransferase, resulting in resistance to lincomycin and lindamycin. All strains carried genes associated with multidrug resistance (*golS*; *mdsA*; *mdsB*; *mdsC*; *mdtK*; *sdiA*; *Mrx*).

### 3.3. Plasmid Replicons and Virulence Genes

Detailed results of the resistant plasmids are shown in Table 4. Three plasmids were detected in two out of eight *Salmonella* strains. In the strain 43_S11 genome, we found plasmids IncHI2_1 and IncHI2A_1, while in isolate 60_S17, we detected plasmid IncL/M(pMU407) 1_pMU407.

In addition, to determine the presence of virulent genes, we analyzed the assembled genomes using VFDB with Abricate. The analysis results showed that all eight isolates carried between 72 and 84 virulence genes and contained 20–24 virulent factors (VFs). Furthermore, all strains carried genes encoding for the invasion of host cells (*InviA-J*). The SPIFinder-2.0 prediction findings indicated the widespread presence of SPI-1, SPI-2, SPI-3, SPI-5, SPI-9, SPI-13, and SPI-14, of which 100% strains had SPI-1, SPI-2, and SPI-3. The most abundant classified serovar was Muenster, with seven out of eight strains belonging to it. Moreover, three out of seven Muenster serovars carried C63PI, an iron transport system in SPI-1.

The mobile element finder (version v.1.0.3, database v.1.0.2) revealed a wide range of plasmid and mobile genetic elements. IncHI2, IncHI2A, and IncL/M are listed among the predicted plasmids (three out of eight strains). The *bla*_CTX-M-55_ gene, which confers resistance to cefotaxime and ceftriaxone, was frequently found in IncHI2.

## 4. Discussion

In this study, 72.34% of whole-duck samples were found to be positive for *Salmonella.* This percentage of contaminated duck carcasses was significantly higher compared to that in previous studies [8,9,17,21,43,44]. According to Zhengquan’s study, the ratio of *Salmonella*-positive results in Southern Chinese retail markets was 41.4% [45], while another study by Li et al. in Sichuan Province (Southwestern Chin) determined that 26.9% of samples at a local market were positive for *Salmonella* [46]. The variety in the *Salmonella* prevalence rate might be attributed to differences in sample location, sample collection time, sampling methods, and *Salmonella* detection methods. However, the outcome of our study on duck carcasses was similar to the results of previous studies that experimented on other types of poultry samples, including chicken. A 2018 study by Zhang et al. in China illustrated the contamination of *Salmonella* in chicken meat at a rate of 63.6% (n = 475) [47]. The *Salmonella*-positive rate was 65.7% (n = 105) in Thailand in 2017 [48] and 2015. In Ho Chi Minh City, Vu et al. reported a 77.63% (n = 76) *Salmonella*-positive rate in chicken meat [49]. In addition, the prevalence of *Salmonella* in our study varied between 69% and 74% and differed in each district. In detail, Ba Dinh had the lowest rate of 69%, while Cau Giay reached the highest amount compared to other districts. This result suggests that the poor hygiene of family-run slaughterhouses might be responsible for the different levels of *Salmonella* in duck meat. Thus, strategies to improve food safety should be implemented to strengthen the supervision of retail markets, improve the market management system (stall sales, tool cleaning, and regular disinfection), and ensure high standards of environmental hygiene (cleaning and drying retail stands) to protect public health.

Our findings revealed that 97.06% (33/34 strains) of whole-strain samples phenotypically expressed resistance to 15 tested antimicrobials. In detail, MDR *Salmonella* was most commonly (28/33 strains, 84.85%) found to have an antimicrobial resistance profile in retail duck meat. This result indicates the significant antibiotic resistance capabilities of *Salmonella* isolates compared to other isolates tested for resistance to *Salmonella* in duck meat. Chen et al., in 2020, also reported that more than 88.1% (133/151 strains) of isolates in duck meat were multidrug-resistant [45]. Based on other published studies on the resistance of *Salmonella*, we found that this rate of duck samples critically surpassed that in other animals including chicken, pork, beef, and shellfish. Van et al. in 2007 demonstrated that 50.5% (n = 18) of *Salmonella* isolates resisted at least one drug, and multidrug resistance was found in all food types [50].

The results of our study correspond to the results of numerous other studies around the world on the antibiotic sensitization of *Salmonella* globally, as reported by Castro-Vargas et al. in 2020. In this author’s research, reports on current multi-resistance were found in 45/46 studies of *Salmonella* in poultry. *Salmonella* strains found in the food chain had high rates of resistance to antibiotics such as nalidixic acid (26.8–86.6%), ampicillin (14.9–68%), ampicillin (14.9–68%), and trimethoprim/sulfamethoxazole (16–54.2%) and were not treatable with carbapenems belonging to families such as imipenem and meropenem [12]. However, our study also showed that the prevalence of *Salmonella* compared to other antibiotics was higher than that reported by Castro-Vargas et al. for cephalosporins belonging to a resistant family (cefazolin, cefuroxime, cefotaxime, ceftazidime, and ceftriaxone), aminoglycosides (gentamicin), and phenicols (chloramphenicol) [13]. Han et al. reported the rate of *Salmonella* isolates from a duck slaughter line (fecal and carcass samples) that resisted ampicillin (59.6%), tetracycline (51.3%), ciprofloxacin (27.6%), ceftriaxone (25.6%), and gentamicin (14.1%) [51]. These results are lower than those obtained in our study.

An important reinforcement of the antibiotic resistance test yielded eight selected strains carrying 43 ARG. All strains carried a variety of aminoglycoside-class ARG (*aac(3)-Iia*, *aac(3)-IId_1*, *aac(6)-Iaa_1*….). Moreover, strain No. 68_S20 carrying the *rmtB* gene encoding for 16S RNA methyltransferase was found to be resistant to all aminoglycoside antibiotic classes, which is an extremely important antibiotic group in animal husbandry and treatment in humans [52]. Furthermore, it is quite surprising that strain 68_S20 with the five beta-lactam family ARG was in the same contig as all eight strains with ESBL phenotypes carrying the *bla*_CTX-M-55_ gene (seven Muenster and one Kentucky serovar) (*bla*_TEM-1B_; *bla*_CTX-M-55_; *bla*_TEM-206_; *bla*_TEM-214_; and *bla*_TEM-141_). Additionally, sample 51_S13 consisted of 11 genes associated with beta-lactam resistance, especially 10/11 genes located in one contig (*bla*_LAP-2_; *bla*_TEM-214_; *bla*_TEM-206_; *bla*_TEM-33_; *bla*_TEM-1B_; *bla*_TEM-216_; *bla*_TEM-209_; *bla*_CTX-M-55_; *bla*_TEM-34_; *bla*_TEM-210_; *bla*_TEM-141_). This is the first report on this gene cluster in Vietnam. The existence of large clusters of genes resistant to antibiotics could help address the potential threat of AMR gene transmission between different strains and species. Notably, analyzing the genomes of eight strains with quinolone antibiotics containing two genes, *floR* and *qnrS1_1,* showed that these genes all carried at least one mutation *parC*:p.T57S. Especially, strain No. 68_S20 carried four mutations (*parC*:p.S80I; *parC*:p.T57S; *gyrA*:p.S83F; and *gyrA*:p.D87N). These mutations resulted in resistance to nalidixic acid and ciprofloxacin and hence could enhance and complicate the quinolone family’s antibiotic resistance; we presume these widely predicted mutations possibly because this group of antibiotics is widely used in agriculture. Another finding of interest in this study was the existence of floR-resistant chloramphenicol and florfenicol. This gene is often based on a mobile genetic factor that exacerbates antibiotic resistance due to transverse, vertical, or variable traits, resulting in the very quick and easy transmission of ARG, even for strains that do not exist under the pressure of that antibiotic. Remarkably, seven genes associated with multidrug resistance (*golS*; *mdsA*; *mdsB*; *mdsC*; *mdtK*; *sdiA*; *Mrx*) were present in all strains.

The WGS data showed that the *Salmonella* serovar Muenster was the dominant serovar isolated in duck carcass, with three different plasmid replicons in *Salmonella* isolates (IncHI2_1, IncHI2A_1, IncL/M(pMU407)_1_pMU407). The plasmid replicons were found to be harbored by *Salmonella* Muenster. Interestingly, IncHI2 and IncHI2A plasmids were harbored by different isolates originating from chickens, ducks, and Muscovy ducks collected from wet markets, which indicated wide dissemination of these plasmids among the other hosts and across distinct geographic regions. These plasmids represent the most significant plasmid lineage implicated in the transmission of antibiotic resistance in *Salmonella*, particularly in *S.* Typhimurium strains. β-lactam (*bla*_OXA-1_ and *bla*_TEM-1_) and quinolone-resistant genes (*qnrA* and *acc(6′)-ib-cr*) were horizontally transferred by the IncHI2 plasmid [53].

In total, 72–84 virulence genes implicated in different mechanisms were recorded using the WGS technique. Notably, our results showed that all eight isolates carried *Salmonella* pathogenicity island 1 (SPI-1) and *Salmonella* pathogenicity island 3 (SPI-3). SPI-1 plays a significant role in the *Salmonella* pathogen by invading epithelial cells. SPI-3 contains the *mgtCB* operon that encodes the MgtC (macrophage survival protein) and MgtB (Mg^2+^ transporter), thereby enhancing the pathogenicity of *Salmonella* [54]. However, these strains contained distinct pathogenic islands, virulent factors, and virulence genes due to the differences in their collection locations.

This study showed that ducks sold in the market are a high source of *Salmonella enterica* infection with very high levels of resistance to many antibiotics and a high diversity of ARG. Therefore, this is a public health issue that deserves public attention.

## Figures and Tables

**Table 1:** Serotyping and cgMLST results.

Sample	Serovar	Serogroup	H1	H2	O Antigen	MLST
31_S7	Muenster	-	e,h	1,5	3,{10}{15}{15,34}	321
42_S10	Muenster	-	e,h	1,5	3,{10}{15}{15,34}	321
43_S11	Muenster	-	e,h	1,5	3,{10}{15}{15,34}	321
45_S12	Muenster	-	e,h	1,5	3,{10}{15}{15,34}	321
51_S13	Muenster	E1	e,h	1,5	3,{10}{15}{15,34}	321
57_S16	Muenster	-	e,h	1,5	3,{10}{15}{15,34}	321
60_S17	Muenster	-	e,h	1,5	3,{10}{15}{15,34}	321
68_S20	Kentucky	C2-C3	i	z6	8,20	198

**Table 2:** Characteristics of sequenced and assembled genomic data.

Sample	Reads	Average Read Length (bp)	Contigs	Genome Length (bp)	Average Contig Length (bp)	N50 (bp)	GC (%)	SRA Accession Number
31_S7	808,752	238	305	4,701,119	129,321	34,942	52.29	SRR21051813
42_S10	683,258	237	425	4,671,790	103,647	28,050	52.45	SRR21051784
43_S11	677,248	238	493	4,905,076	101,196	23,437	52.15	SRR21051783
45_S12	749,140	239	334	4,744,687	160,345	37,074	52.27	SRR21051781
51_S13	783,124	239	329	4,766,796	157,729	36,997	52.32	SRR21051780
57_S16	591,734	237	382	4,655,893	156,597	30,153	52.41	SRR21051777
60_S17	655,310	238	493	4,682,797	87,054	22,011	52.51	SRR21051776
68_S20	910,644	237	464	4,804,148	181,020	24,436	52.47	SRR21051782

**Table 3:** Distribution of ARG in *Salmonella* serovars based on in silico predictions.

			Samples								
			68_S20	43_S11	45_S12	51_S13	31_S7	42_S10	57_S16	60_S17	
		Serovar	Kentucky	Muenster	Muenster	Muenster	Muenster	Muenster	Muenster	Muenster	
		Genes/Number of genes identified	33	27	26	33	25	26	26	24	
Drug classes	Rifampin	*arr-3_4*	1	1	1	1	1	1	1	1	
		*arr2*	1	1	1	1	1	1	1	1	
	Aminoglycoside	*aac(3)-Iia*	1	1	1	1	1	1	1	1	
		*aac(3)-IId_1*	1	1	1	1	1	1	1	1	
		*aac(3)-Id_1*	1								Absence (Negative)
		*aac(6)-Iaa_1*	1	1	1	1	1	1	1	1	
		*aac(6)-Iy*		1	1	1	1	1	1	1	
		*aadA17*	1								
		*aadA22*		1	1	1	1	1	1	1	
		*aadA7_1*	1								
		*ant(3)-Ia_1*	1	1	1	1	1	1	1	1	
		*aph(3)-Ia_3*	1	1					1	1	
		*aph(6)-Id_1*		1	1	1	1	1	1		
		*rmtB_1*	1								
	Beta-lactam	*bla*_CTX-M-55_1_	1	1	1	1	1	1	1	1	
		*bla*_LAP-2_		1	1	1	1	1		1	
		*bla*_TEM-141_	1			1					
		*bla*_TEM-1B_1_	1	1	1	1	1	1	1	1	
		*bla*_TEM-206_	1			1					
		*bla*_TEM-209_				1					
		*bla*_TEM-210_				1					
		*bla*_TEM-214_	1			1					
		*bla*_TEM-216_				1					
		*bla*_TEM-33_				1					presence (Positive)
		*bla*_TEM-34_				1					
	Fosfomycin	*fosA3_1*	1								
	Chloramphenicol	*floR_2*	1	1	1			1	1	1	
	Diaminopyrimidine	*dfrA14_5*	1	1	1	1	1	1	1	1	
	Lincosamide	*linG*	1	1	1	1	1	1	1	1	
		*Inu(F)_1*	1	1	1	1	1	1	1	1	
	Quinolone	*qnrS1_1*	1	1	1	1	1	1	1	1	
	Macrolides	*mph(A)-2*	1								
	Sulfonamides	*sul1_5*	1								
		*sul3_2*		1	1	1	1	1	1	1	
	Tetracyclin	*tet(A)_6*	1	1	1	1	1	1	1		
		*tetR*	1	1	1	1	1	1	1		
	Multi-drug classes	*golS*	1	1	1	1	1	1	1	1	
		*mdsA*	1	1	1	1	1	1	1	1	
		*mdsB*	1	1	1	1	1	1	1	1	
		*mdsC*	1	1	1	1	1	1	1	1	
		*mdtK*	1	1	1	1	1	1	1	1	
		*Mrx*	1								
		*sdiA*	1	1	1	1	1	1	1	1	

**Table 4:** Plasmids, virulence factors, and SPI results.

Strains	Serotype	Plasmid	Number of Virulence Factors	Number of Virulence Genes	SPI
31_S7	Muenster		24	82	SPI-1, SPI-2, SPI-3, SPI-5, SPI-9, SPI-13, SPI-14
42_S10	Muenster		23	79	C63PI, SPI-1, SPI-2, SPI-3, SPI-5, SPI-9, SPI-13, SPI-14
43_S11	Muenster	IncHI2_1 IncHI2A_1	20	75	Not_named, SPI-1, SPI-2, SPI-3, SPI-9, SPI-13, SPI-14
45_S12	Muenster		21	79	SPI-1, SPI-2, SPI-3, SPI-5, SPI-13, SPI-14
51_S13	Muenster		23	84	C63PI, SPI-1, SPI-2, SPI-3, SPI-5, SPI-9, SPI-13
57_S16	Muenster		23	81	C63PI, SPI-1, SPI-2, SPI-3, SPI-13
60_S17	Muenster	IncL/M(pMU407)_1_pMU407	21	72	Not_named, SPI-1, SPI-2, SPI-3, SPI-13, SPI-14
68_S20	Kentucky		23	83	SPI-1, SPI-2, SPI-3, SPI-9

## Data Availability

Not applicable.

## Appendix A

**Table A1:** Antibiotic resistance profiles of *Salmonella* isolates (zone of inhibition expressed in millimeters).

Isolation of Samples	CXM (mm)	CRO (mm)	FOX (mm)	CZ (mm)	CTX (mm)	CAZ (mm)	TM (mm)	TE (mm)	C (mm)	CN (mm)	NA (mm)	CIP (mm)	AMP (mm)	MRP (mm)	Resistance Number
Ba Đinh District	R (8)	R (6)	S (25)	R (6)	R (6)	R (6)	R (6)	R (8)	S (26)	R (6)	I (18)	S (27)	R (6)	S (35)	9
	S (22)	I (21)	S (24)	I (20)	S (33)	S (25)	S (33)	I (12)	R (8)	S (20)	S (24)	S (39)	S (21)	S (37)	1
	R (9)	R (6)	S (24)	R (6)	R (6)	R (6)	R (6)	R (11)	R (6)	R (6)	S (20)	S (31)	R (6)	S (35)	10
	R (6)	R (9)	S (27)	R (6)	R (6)	R (6)	R (6)	R (6)	R (6)	S (12)	I (17)	S (25)	R (6)	S (30)	10
	S (27)	S (32)	S (27)	S (23)	S (38)	S (27)	S (32)	S (18)	S (30)	S (20)	I (18)	I (28)	S (21)	S (25)	0
Cau Giay District	R (10)	R (10)	S (21)	R (6)	R (8)	R (13)	R (6)	R (6)	R (6)	R (12)	R (6)	S (30)	R (6)	S (34)	11
	R (10)	R (6)	S (18)	R (6)	R (6)	R (6)	R (6)	R (6)	R (6)	R (6)	R (12)	S (27)	R (6)	S (37)	11
	R (6)	R (6)	S (26)	R (6)	R (6)	R (6)	R (6)	R (10)	R (6)	R (6)	S (20)	S (35)	R (6)	S (35)	10
Dong Da District	S (22)	S (34)	S (23)	I (21)	S (31)	S (23)	R (6)	R (11)	R (6)	R (6)	S (21)	S (29)	S (19)	S (32)	4
	R (6)	R (6)	S (25)	R (6)	R (6)	R (6)	R (6)	R (6)	S (25)	R (8)	I (17)	I (23)	R (6)	S (30)	9
	S (26)	S (37)	S (28)	I (21)	S (34)	S (27)	S (33)	R (11)	S (30)	S (21)	S (27)	S (41)	S (21)	S (26)	1
	R (6)	R (8)	S (19)	R (6)	R (10)	I (20)	R (6)	R (6)	R (6)	R (11)	R (6)	S (30)	R (6)	S (32)	10
	R (6)	R (11)	S (25)	R (6)	R (16)	S (22)	R (6)	R (6)	R (6)	R (12)	R (6)	S (32)	R (6)	S (35)	10
	R (6)	R (12)	S (23)	R (6)	R (19)	S (27)	R (6)	R (6)	R (6)	R (11)	R (6)	S (31)	R (6)	S (35)	10
	R (6)	R (8)	S (29)	R (6)	R (7)	I (19)	R (6)	R (6)	R (6)	R (10)	R (6)	S (34)	R (6)	S (37)	10
	R (6)	R (12)	S (25)	R (6)	R (19)	S (21)	S (30)	R (6)	R (6)	R (12)	R (6)	S (32)	R (6)	S (35)	9
	R (6)	R (9)	I (17)	R (6)	R (9)	R (16)	S (28)	R (6)	R (6)	R (10)	R (6)	S (27)	R (6)	S (31)	10
Hoang Mai District	S (22)	S (32)	S (24)	R (17)	S (34)	S (25)	R (6)	R (8)	S (27)	S (20)	I (15)	S (34)	R (6)	S (35)	4
	R (6)	R (6)	S (24)	R (6)	R (6)	R (15)	R (6)	R (11)	R (6)	R (9)	R (6)	R (13)	R (6)	S (35)	10
	R (6)	R (8)	S (21)	R (6)	R (8)	R (12)	R (6)	R (6)	R (6)	R	R (6)	R (8)	R (6)	S (36)	10
	R (6)	R (6)	S (33)	R (6)	R (8)	S (26)	R (6)	I (12)	R (6)	R (6)	R (6)	R (18)	R (6)	S (30)	8
	R (6)	R (8)	S (21)	R (6)	R (9)	S (21)	R (6)	R (6)	R (6)	R (12)	R (6)	S (32)	R (6)	S (36)	9
	S (21)	S (35)	S (19)	R (15)	S (36)	S (25)	R (6)	R (8)	R (6)	S (17)	R (11)	I (23)	R (6)	S (30)	5
	S (25)	S (38)	S (27)	I (21)	S (39)	S (27)	S (36)	R (10)	S (32)	S (22)	I (16)	S (34)	S (22)	S (26)	1
	S (22)	S (33)	S (25)	I (20)	S (35)	S (27)	R (6)	I (14)	S (29)	S (20)	I (15)	S (28)	S (22)	S (23)	1
Thanh Xuan District	S (21)	S (31)	S (23)	R (17)	S (32)	I (20)	R (6)	R (8)	R (6)	S (17)	I (15)	S (30)	R (6)	S (30)	5
	R (9)	R (9)	S (23)	R (6)	R (9)	R (13)	R (6)	R (11)	S (24)	R (12)	I (15)	I (24)	R (6)	S (29)	9
	R (6)	R (6)	S (20)	R (6)	R (6)	R (6)	R (6)	R (6)	R (6)	R (6)	R (12)	I (25)	R (6)	S (34)	11
	R (10)	R (6)	S (21)	R (6)	R (6)	R (6)	R (6)	I (14)	R (6)	R (6)	R (6)	S (26)	R (6)	S (37)	10
	S (22)	S (31)	S (24)	R (17)	S (33)	S (23)	R (6)	R (8)	R (6)	S (17)	R (11)	I (23)	R (6)	S (30)	6
	R (6)	R (11)	S (30)	R (6)	R (9)	R (6)	R (6)	R (10)	R (6)	R (6)	S (20)	S (30)	R (6)	S (35)	10
	R (6)	R (13)	S (23)	R (6)	R (12)	I (20)	R (6)	R (6)	R (6)	R (12)	R (6)	S (29)	R (6)	S (32)	10
	R (6)	R (9)	S (20)	R (6)	R (13)	I (19)	S (28)	R (11)	R (6)	S (20)	R (6)	S (31)	R (6)	S (33)	8
	S (20)	S (31)	S (20)	R (18)	S (30)	S (23)	S (30)	S (19)	S (27)	S (19)	S (22)	S (36)	S (18)	S (35)	1
*E. coli* ATCC 25922 (negative control)	S (28)	S (27)	S (26)	S (34)	S (35)	S (29)	S (30)	S (30)	S (32)	S (30)	S (29)	S (31)	S (32)	S (30)	-

**Table A2:** Antimicrobial resistance genes in *Salmonella* isolates.

			Drug Classes												
Antibiotic Resistance	Code Strain	Strains	Aminoglycoside	Beta-Lactam	Chloramphenicol	Quinolone	Macrolides	Tetracycline	Sulfonamides	Fosfomycin	Diaminopyrimidine	Rifampin	Lincosamide	Polypeptide	Multidrug Classes
CMX-CRO-CZ-CTX-CAZ-TM-CN-TE-AMP	68_S20	Kentucky	*aac(3)-Iia*; *aac(3)-Id*; *aac(3)-IId_1*; *aac(6)-Iaa_1*; *aadA17*; *aadA7_1*; *ant(3)-Ia_1*; *aph(3)-Ia_3*; *rmtB_1*;	*bla*_CTX-M-55_; *bla*_TEM-206_; *bla*_TEM-1B_; *bla*_TEM-141_; *bla*_TEM-214_	*floR_2*	*qnrS1_1*	*mph(A)-2*	*Tet(A)_6*; *TetR*	*sul1_5*;	*fosA3_1*	*dfrA14_5*	*ARR-3_4*; *ARR-2*;	*lnu(F)_1*; *linG*;		*golS*; *mdsA*; *mdsB*; *mdsC*; *mdtK*; *sdiA*; *Mrx*;
CMX-CRO-CZ-CTX-CAZ-TM-CN-TE-C-AMP	43_S11	Muenster	*aac(3)-Iia*; *aac(3)-IId_1*; *aac(6)-Iaa_1*; *aac(6)-Iy*; *aadA22*; *ant(3)-Ia_1*; *aph(3)-Ia_3*; *aph(6)-Id_1*;	*bla*_CTX-M-55_; *bla*_TEM-1B_1_; *bla*_LAP-2_	*floR_2*	*qnrS1_1*		*Tet(A)_6*; *TetR*	*sul3_2*;		*dfrA14_5*	*ARR-3_4*; *ARR-2*;	*lnu(F)_1*; *linG*;		*golS*; *mdsA*; *mdsB*; *mdsC*; *mdtK*; *sdiA*;
CMX-CRO-CZ-CTX-CAZ-TM-CN-TE-C-AMP	45_S12	Muenster	*aac(3)-Iia*; *aac(3)-IId_1*; *aac(6)-Iaa_1*; *aac(6)-Iy*; *aadA22*; *ant(3)-Ia_1*; *aph(6)-Id_1*;	*bla*_CTX-M-55_; *bla*_TEM-1B_1_; *bla*_LAP-2_	*floR_2*	*qnrS1_1*		*Tet(A)_6*; *TetR*	*sul3_2*;		*dfrA14_5*	*ARR-3_4*; *ARR-2*;	*lnu(F)_1*; *linG*;		*golS*; *mdsA*; *mdsB*; *mdsC*; *mdtK*; *sdiA*;
CMX-CRO-CZ-CTX-CAZ-TM-CN-TE-C-AMP	51_S13	Muenster	*aac(3)-Iia*; *aac(3)-IId_1*; *aac(6)-Iaa_1*; *aac(6)-Iy*; *aadA22*; *ant(3)-Ia_1*; *aph(6)-Id_1*;	*bla*_CTX-M-55_; *bla*_TEM-1B_1_; *bla*_LAP-2_; *bla*_TEM-214_; *bla*_TEM-206_; *bla*_TEM-33_; *bla*_TEM-216_; *bla*_TEM-209_; *bla*_TEM-34_; *bla*_TEM-210_; *bla*_TEM-141_		*qnrS1_1*		*Tet(A)_6*; *TetR*	*sul3_2*;		*dfrA14_5*	*ARR-3_4*; *ARR-2*;	*lnu(F)_1*; *linG*;		*golS*; *mdsA*; *mdsB*; *mdsC*; *mdtK*; *sdiA*;
CMX-CRO-CZ-CTX-CAZ-TM-CN-TE-AMP	31_S7	Muenster	*aac(3)-Iia*; *aac(3)-IId_1*; *aac(6)-Iaa_1*; *aac(6)-Iy*; *aadA22*; *ant(3)-Ia_1*; *aph(6)-Id_1*;	*bla*_CTX-M-55_; *bla*_TEM-1B_1_; *bla*_LAP-2_		*qnrS1_1*		*Tet(A)_6*; *TetR*	*sul3_2*;		*dfrA14_5*	*ARR-3_4*; *ARR-2*;	*lnu(F)_1*; *linG*;		*golS*; *mdsA*; *mdsB*; *mdsC*; *mdtK*; *sdiA*;
CMX-CRO-CZ-CTX-CAZ-TM-CN-TE-C-AMP	42_S10	Muenster	*aac(3)-Iia*; *aac(3)-IId_1*; *aac(6)-Iaa_1*; *aac(6)-Iy*; *aadA22*; *ant(3)-Ia_1*; *aph(6)-Id_1*;	*bla*_CTX-M-55_; *bla*_TEM-1B_1_; *bla*_LAP-2_	*floR_2*	*qnrS1_1*		*Tet(A)_6*; *TetR*	*sul3_2*;		*dfrA14_5*	*ARR-3_4*; *ARR-2*;	*lnu(F)_1*; *linG*;		*golS*; *mdsA*; *mdsB*; *mdsC*; *mdtK*; *sdiA*;
CMX-CRO-CZ-CTX-CAZ-TM-CN-C-AMP	57_S16	Muenster	*aac(3)-Iia*; *aac(3)-IId_1*; *aac(6)-Iaa_1*; *aac(6)-Iy*; *aadA22*; *ant(3)-Ia_1*; *aph(3)-Ia_3*; *aph(6)-Id_1*;	*bla*_CTX-M-55_; *bla*_TEM-1B_1_	*floR_2*	*qnrS1_1*		*Tet(A)_6*; *TetR*	*sul3_2*;		*dfrA14_5*	*ARR-3_4*; *ARR-2*;	*lnu(F)_1*; *linG*;		*golS*; *mdsA*; *mdsB*; *mdsC*; *mdtK*; *sdiA*;
CMX-CRO-CZ-CTX-CAZ-TM-CN-TE-C-AMP	60_S17	Muenster	*aac(3)-Iia*; *aac(3)-IId_1*; *aac(6)-Iaa_1*; *aac(6)-Iy*; *aadA22*; *ant(3)-Ia_1*; *aph(3)-Ia_3*;	*bla*_CTX-M-55_; *bla*_TEM-1B_1_; *bla*_LAP-2_	*floR_2*	*qnrS1_1*			*sul3_2*;		*dfrA14_5*	*ARR-3_4*; *ARR-2*;	*lnu(F)_1*; *linG*;		*golS*; *mdsA*; *mdsB*; *mdsC*; *mdtK*; *sdiA*;
